# Aortic aneurysm sac filling with AneuFix injectable polymer during endovascular aneurysm repair: feasibility and safety trial study protocol

**DOI:** 10.1136/bmjopen-2023-082380

**Published:** 2024-07-15

**Authors:** Stefan P M Smorenburg, Rutger J Lely, Michael J Jacobs, Arjan W J Hoksbergen

**Affiliations:** 1 Department of Surgery, Amsterdam UMC location Vrije Universiteit, Amsterdam, The Netherlands; 2 Amsterdam Cardiovascular Sciences, Amsterdam, The Netherlands; 3 Department of Radiology, Amsterdam UMC location Vrije Universiteit, Amsterdam, The Netherlands; 4 Department of Vascular Surgery, European Vascular Centre Maastricht-Aachen, Maastricht University Medical Centre, Maastricht, The Netherlands

**Keywords:** Vascular surgery, Interventional radiology, Feasibility Studies, Clinical trials

## Abstract

**Introduction:**

Type II endoleaks (T2ELs) following endovascular aneurysm repair (EVAR) for abdominal aortic aneurysm (AAA) can lead to aneurysm growth, compromising the stent graft seal and risking rupture. Preventing these endoleaks during EVAR involves filling the AAA sac around the stent graft to exclude the aneurysm and block any arteries causing the endoleak. This study investigates the feasibility and safety of using AneuFix, a biocompatible injectable polymer developed by TripleMed (Geleen, the Netherlands), for aneurysmal sac filling during EVAR in high-risk T2EL patients.

**Methods and analysis:**

A feasibility, single-arm, single-centre clinical trial will initially include five patients with infrarenal AAA, eligible for EVAR, and at high risk for T2EL based on the number of patent lumbar arteries and the cross-sectional area of the aortic lumen at the level of the inferior mesenteric artery. Postevaluation by the Data Safety and Monitoring Board, the study cohort will extend to 25 patients. During EVAR and after stent graft deployment, the aneurysm sac is filled with AneuFix polymer using a filling sheath positioned parallel to the contralateral limb with the tip inside the aneurysm sac. Primary outcome is technical success (successful AAA sac filling). The secondary outcomes include clinical success at 6 and 12 months (occurrence of T2ELs and AAA growth assessed with CT angiography), intraoperative and perioperative complications, all endoleaks, adverse events, re-interventions, aneurysm rupture and patient survival.

**Ethics and dissemination:**

This trial was approved by the Dutch Authorities (Central Committee on Research Involving Human Subjects, IGJ), Amsterdam University Medical Centre Ethical Commission, and adheres to the Declaration of Helsinki and European Medical Device Regulation. Results will be shared at (inter)national conferences and in peer-reviewed journals.

**Trial registration number:**

NCT04307992.

**STRENGTHS AND LIMITATIONS OF THIS STUDY**

- AneuFix polymer is designed to be an easy-to-administer material for filling the abdominal aortic aneurysm lumen around the stent-graft during endovascular aneurysm repair.
- The injectable polymer can prevent type II endoleaks with a short additional procedure time.
- The feasibility trial contains no control group and the sample size is small.

## Introduction

Type II endoleak (T2EL) is caused by a retrograde blood flow from lumbar arteries or the inferior mesenteric artery (IMA) into the aortic abdominal aneurysm (AAA) sac after endovascular aneurysm repair (EVAR). It occurs in 18%–30% patients treated with EVAR.^1^,^5^ A large fraction will resolve spontaneously over time however 15%–35%^4^,^6^ of the patients with persistent T2EL are at risk for continuous AAA growth which can compromise aortic seal zones (proximal or distal) and might eventually cause aortic rupture in 2%.^4^

## Background and relevant literature and data

In order to prevent T2EL, several AAA sac management strategies are being developed and implemented worldwide. Pre-emptive embolisation of aneurysm sac side branches with coils can be effective, but requires a significant learning curve, requires an additional procedure and is time consuming.^6^,^10^ Another strategy is intraoperative prophylactic AAA sac filling. The aneurysm sac can be filled with an embolisation material (coils and/or liquid) during EVAR after which the aneurysm sac is embolised.^11^,^17^ Furthermore, endovascular aneurysm sealing (EVAS) has been, for a short period of time in the past, widely performed, however, a device recall decreased the implantations significantly.^18^

The crucial difference between EVAR with active sac filling and EVAS is the approach to sealing the aneurysm and stent-graft fixation. EVAS does not involve fixation above or below the aneurysm. Instead, it focuses on sealing off the aneurysm sac itself using polymer-filled endobags that conform to the sac shape. In contrast, EVAR with active sac filling uses a standard EVAR stent graft, which achieves proximal and distal seal above and below the aneurysm in relatively healthy aortoiliac tissue, effectively isolating the aneurysm from blood flow. Adjacent to this, the AAA sac is filled around the standard EVAR stent graft.

In recent years, a novel embolic material was developed specifically for AAA sac filling during EVAR; AneuFix (TripleMed, Geleen, the Netherlands) injectable polymer.

In this study, we will investigate the feasibility and safety of AneuFix use for aneurysm sac filling during EVAR in patients with high risk of T2EL.

## Methods and analysis

### AneuFix biocompatible injectable polymer

AneuFix is a two-component injectable polymer, based on polydimethylsiloxane which is widely applied for long-term implants such as dental and bone because of its biological inertness and stability.^19 20^ It is a medical implant which has the unique properties to change viscosity during the procedure, from low viscosity (tooth-paste like) during injection to high viscosity after injection and after 2 min cures to a flexible, solid and compliant implant. AneuFix has been extensively studied in preclinical tests and demonstrated to be biocompatible, biostable and has a thrombogenicity comparable to expanded polytetrafluorethylene; a material used widely in current aortic stentgrafts.^20 21^ Before clinical application, to prove feasibility and safety several in vitro and in vivo experimental studies with AneuFix have been conducted and published.^20^,^25^ Recently, we published our initial experience and short-term results of T2EL treatment with AneuFix, for patients with AAA growth with T2EL after EVAR during follow-up.^26^

**Figure 1:**
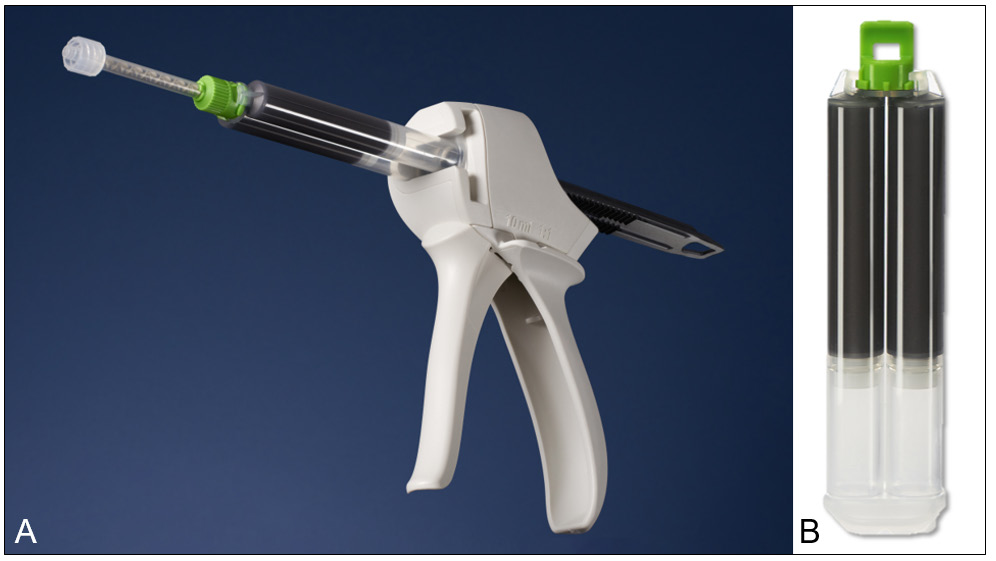
Dispenser for injection with static mixer (**A**) and syringe with AneuFix material (**B**).

The AneuFix-kit contains a double-barrel 2×20 mL syringe with the polymer, a dispenser gun and a static mixer which is mounted onto the dispenser (figure 1A,B). During injection, both components are mixed in the static mixer through a plastic helix, after which the curing process starts. AneuFix contains 30% tantalum, to ensure real-time visualisation under fluoroscopic guidance.

The implant was classified as a class III medical device. In the European Union, these medical devices require CE certification by the notified body before introduction into the market. AneuFix is pre-CE mark and will go for registration after enough patients are treated during the pilot phase.

### Study objectives

To investigate the safety and feasibility of prophylactic aortic aneurysm sac filling with AneuFix injectable polymer during EVAR in patients at high risk for T2EL, as defined in the inclusion criteria.

### Study design

**Figure 2:**
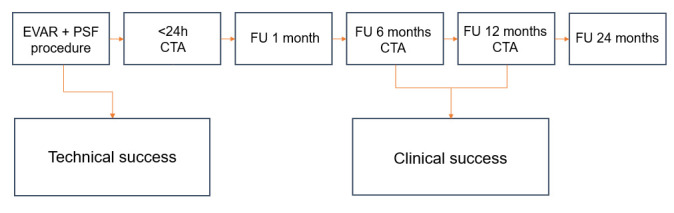
Overview of study follow-up. CTA, CT angiography; EVAR, endovascular aneurysm repair; FU, follow-up; PSF, prophylactic sac filling.

This is a feasibility, single-arm, single-centre clinical trial. Initially, a pilot study will be performed with five patients. The Data Safety and Monitoring Board (DSMB) will extensively evaluate the procedure imaging, clinical data and outcome after the second, fourth and fifth patients are treated. If safety and feasibility are confirmed by the DSMB, the total cohort will be expanded to 25 patients. After the pilot study and subsequent DSMB evaluation, the remaining 20 patients will be treated. Figure 2 displays the study design.

### Primary endpoint

#### Technical success

Technical success defined as successful AAA sac filling during EVAR.

### Primary safety endpoints

#### Adverse events

Incidence of (serious) adverse events (SAEs) and adverse device effects, both intraoperatively and during follow-up, related to AneuFix and the procedure (online supplemental material 1 for full definitions).

#### Perioperative complications

The rate of any complications occurring within the first 30 days post-EVAR with AneuFix.

#### Aneurysm rupture

The rate of aneurysm rupture occurring during the inclusion period.

#### Patient survival

The proportion of patients surviving, and overall clinical outcome, assessed throughout the study up to 24 months.

### Secondary endpoints

#### Clinical success

Clinical success defined as the occurrence of T2ELs and aneurysmal sac growth, as assessed at both 6 and 12 months following the EVAR procedure, and confirmed through CT angiography (CTA).

#### Intraoperative parameters

Intraoperative parameters including total AneuFix volume injected, injection time, proximal balloon inflation time, procedure time, blood loss, total contrast volume used, fluoroscopy time, total radiation exposure.

#### All endoleaks

Occurrence of any type of endoleaks (I–IV) during the complete inclusion period as confirmed on CTA imaging.

#### Re-interventions

The rate of secondary endovascular or surgical re-interventions, including the rate of conversion to open AAA repair.

### Study population

Patients who are indicated for EVAR to treat infrarenal AAA.

### Inclusion criteria

Patients with infrarenal AAA and high-risk profile for developing endoleak type II, as per recommendations of Güntner *et al*,^27^ including:

- Open IMA, and:
  - At least one patent lumbar artery with cross-sectional area at the location of the IMA (CSA^IMA^)>17.5 cm², or
  - two patent lumbar arteries and CSA^IMA^>15 cm², or
  - three patent lumbar arteries and CSA^IMA^>12.5 cm², or
  - four patent lumbar arteries and CSA^IMA^>10 cm², or
  - five patent lumbar arteries and CSA^IMA^>7.5 cm².

Furthermore, patients should have an infrarenal aortic neck which is compliant with the instructions for use (IFU) of the EVAR device and a suitable aortic-iliac anatomical configuration for EVAR, as per the criteria of the selected EVAR device. Additionally, patients must have a life expectancy of at least 2 years and be older than 18 years. Finally, they must demonstrate willingness and ability to comply with the clinical study requirements.

### Exclusion criteria

Exclusion criteria for the study include patients who are not able or willing to give written informed consent, those undergoing emergency procedures, or those undergoing EVAR for ruptured or symptomatic AAA. Patients with a suprarenal AAA, an inflammatory AAA with more than minimal wall thickening or an infrarenal neck unsuitable for endovascular fixation (including so-called ‘hostile necks’) are also excluded. This also applies to those with an aortic-iliac anatomical configuration otherwise unsuitable for EVAR according to the criteria of the device to be used. Patients with evidence of a type Ia or type III endoleak persistent after balloon inflation on the completion angiogram during EVAR, those in whom a bilateral retroperitoneal incision or the sacrifice of both hypogastric arteries is required for EVAR, and those with anatomical variations such as a horseshoe kidney are not eligible. Additional exclusions include patients who cannot be administered contrast agents due to a proven severe systemic reaction, those with an active infection, patients scheduled for or having received an organ transplant, and those with a limited life expectancy due to other illnesses (less than 1 year). Patients with non-iatrogenic bleeding diathesis, women of childbearing potential, and those with connective tissue disease are also not eligible for the study.

### Procedure steps

#### Standard EVAR with filling sheath

The stent graft manufacturer’s IFU should be followed during preoperative stent graft sizing. The procedure starts like a standard EVAR; the patient is positioned supine. Intra-arterial access can be gained by either groin cut down or percutaneous puncture according to physicians preference. AneuFix polymer is administered through a 7F, 45 cm sheath that is advanced through an 18F, 13 cm access sheath in the groin on the filling side (right or left) of the patient. The regular EVAR steps should be followed, up until the deployment of the contralateral stent graft limb. The contralateral limb should be inserted and not yet deployed. A guidewire should be placed in the AAA sac, functioning as guidance for the filling sheath. After contralateral limb deployment the guidewire is jailed between the outside of the stent graft leg and iliac aneurysm wall. The 7F filling sheath should be advanced over the jailed guidewire, into the aneurysm sac with the tip inside the aneurysm sac. Then, ballooning of the proximal and distal seal zones is performed. Subsequently, a completion angiography is performed with a diagnostic pigtail catheter at the level of the renal arteries to determine patency of the EVAR stent graft and presence of any endoleak.

#### Aneurysm sac filling with AneuFix

First, a moulding balloon is introduced and placed within the proximal seal zone. This balloon should be inflated during AneuFix injection to diminish the remaining blood flow and pressure within the aneurysm sac caused by collateral flow and lumbar arteries. Then, an initial saccogram is made through the filling sheath inside the aneurysm sac to determine: endoleak nidus, location and amount of lumbar arteries and location of the IMA. Also when present, the location of a potential Adamkiewicz artery arising from the infrarenal aorta should be visualised. The contours of the sac angiography should be delineated, ensuring the borders of the endoleak nidus and AAA thrombus are clearly defined.

When all previously stated arteries are located, the C-arm should be adjusted to a lateral position, to visualise the ostia and trajectory of the lumbar arteries and the IMA. The tip of the filling sheath should be placed near the proximal stent graft landing zone ensuring a pull-back motion during injection.

The next step is preparation for AneuFix injection: the static mixer is placed on the syringe and the syringe is placed in the dispenser. A three-way stopcock, primed with saline is connected to the end of the static mixer. Air is squeezed out from the syringe by holding it vertically upright, after which the static mixer is filled with AneuFix. Squeeze out 1–2 mL of polymer to assure the polymer is properly mixed. Connect the static mixer syringe via the Luer connection to the filling sheath and the polymer is ready for injection.

Start dilation of the balloon in the proximal seal zone of the stent graft and start AneuFix injection: slowly but steadily inject AneuFix under visual control into the aneurysm sac around the proximal part of the stent graft, move the filling sheath slowly distal during injection. The lumbar arteries and IMA should be closely monitored. If needed, replace the first syringe by a second or third, depending on the total volume to be filled.

When the polymer has reached the most caudal part of the aneurysm and a small segment of the lumbar arteries and AMI have been filled, the AneuFix injection can be stopped. The balloon in the proximal seal zone of the stent graft is deflated.

The visual control of filling the complete sac and continuous visibility of the IMA/lumbar artery ostia is critical in order to prevent injection of AneuFix too far distal in these arteries. Also, in order to prevent a polymer gutter along the stent graft limb, a dilatation balloon may be inflated inside the distal seal zone of the stent graft limb at the end of sac filling, to further minimise the chance of polymer gutter along the stent graft limb. When the sac fill is complete and before retracting the filling sheath into the access sheath, ensure that the AneuFix material within the filling sheath is drawn back by applying a vacuum to the sheath with a 20 mL syringe.

After complete sac filling, an angiogram with a diagnostic pigtail catheter placed just above the renal arteries is performed to visualise the EVAR stent graft and AneuFix polymer within the aneurysm sac. After this, the remaining guidewires and catheters are removed. Optionally, a blank cone-beam CT can be made to assess EVAR stent graft geometry and polymer placement in 3D around the stent graft. The procedure is ended by closing the arterial access sites and groin closing.

### Assessments and follow-up

#### Screening assessments

Demographic data, relevant medical history, comorbidities are gathered from the electronic patient record file. The AAA volume and sac anatomy are determined from preoperative CTA (<90 days old). Relevant data on current medication, estimated glomerular filtration rate and serum creatinine is gathered. If all the inclusion and none of the exclusion criteria are met, patients will be considered for the procedure.

Furthermore, according to the protocol inclusion criteria (version 1.7), all patients are screened for high risk of T2EL development; the amount of patent lumbar arteries is measured on preoperative CTA and the CSA of the aortic lumen at the level of the IMA.

#### Baseline assessments

The baseline assessments are performed on the day of hospital submission, before EVAR and AneuFix injection procedure: relevant current medication, C-reactive protein, adverse events since screening visit.

#### Postprocedure until hospital discharge

Within the first 24 hours, and prior to patient discharge, CTA is performed to evaluate the appropriate filling of the aneurysm sac and absence of T2ELs. Adverse events are recorded if present. Also, postprocedural medication is recorded, C reactive protein is assessed.

#### 1-month follow-up

A telephone call or outpatient clinic visit (dependent on physician and patient preference) is done to record any adverse events and record relevant current medication at 1-month follow-up (±10 days).

#### 6-months follow-up

CTA imaging (blank, arterial, venous with scatter reduction) is performed to determine clinical success at 6-months follow-up ±30 days. Any medication change will be registered and any adverse events.

#### 12-months follow-up

CTA imaging (blank, arterial, venous with scatter reduction) is performed to determine clinical success at 12-months follow-up (±30 days). Any medication change will be registered and any adverse events.

#### 24-months follow-up

Two years after AneuFix injection, patients will be followed up by telephone call (±30 days). The intention of this phone call is to assess any adverse effects that may have occurred since the previous last follow-up visit, potential re-admissions and to assess patient survival.

### Study exit

After the 24-month follow-up, patients have reached study completion however, patients will remain in follow-up per local hospital protocol typically involving yearly duplex or CT assessment.

### Statistical analysis

#### Randomisation and stratification

No randomisation will occur, enrolled patients are intended to get sac filling with AneuFix.

#### Data analysis and presentation

Normal distributed continuous outcome variables will be summarised by their mean and SD, categorical variables will be summarised as proportions. Non-normal distributed continuous outcome variables will be summarised by their median and IQR. Study results will be reported in the final study report in a coded manner and in accordance to the current European Union General Data Protection Regulation (GDPR). Data will be available on request.

#### Statistical analysis of primary endpoints

The outcome of all treated patients will be represented as a proportion of technical success. For safety, the ASEs related to the procedure or device will be reported.

#### Sample size determination

First, a pilot study will be conducted with five patients. After DSMB approval, this sample size will be raised to 25 patients by treating another 20 patients. A sample size of 25 has been calculated adequate and sufficient to assess the goals of the study with an independent statistician and instructed by the CE-notified body.

#### Study runtime

The study will run from 15 February 2022 until 13 December 2025.

### Patient and public involvement

No patients were involved in the design of this study.

## Ethics and dissemination

The study is executed in line with the relevant articles of the Declaration of Helsinki as adopted by the 18th World Medical Assembly in 1964 and as revised repeatedly on 19 October 2013. In the Netherlands, the Medical Research Involving Human Subjects Act (WMO) guides the implementation of this study protocol.

Formal ethics committee approval prior to the start of the study must be obtained prior to enrolment of any patient. The documents which are delivered to the investigator for this purpose are: Clinical Investigational Plan (CIP version 1.7), Investigator Brochure, Participant Information Sheet and Informed Consent Form provided in local language, Clinical Trial Agreement specifying the proposed compensation for study execution, evidence of clinical investigation insurance. Significant amendments to the protocol and the informed consent are to be approved by the ethics committee prior implementation. Patients were not involved in the study design. We used the Standard Protocol Items: Recommendations for Interventional Trials checklist when writing our report.^28^

The use of investigational devices also requires the National Competent Authority approval prior to the start of the study. The sponsor has obtained the approval prior to implementation of the study in any of the investigational sites.

Each patient will be appropriately informed about the study purpose and their voluntary participation will be documented in writing by signing the provided informed consent form.

A patient specific insurance is subscribed where the study is conducted. This insurance for the patients is in line with article 7 of the WMO and is adequate to cover for incidences related to the conduct of the clinical trial and that become apparent during the study or within 4 years after the end of the study.

This clinical investigation is executed in accordance with the ISO 14155 (2011). The definitions of AEs listed in this ISO-standard apply to the study. All vascular related adverse events; ADEs, SAEs, SADEs and USADEs throughout the clinical investigation shall be documented in a timely manner.

The DSMB is an independent group consisting of expert clinicians, who collectively have experience in the management of patients with abdominal aneurysm treatment and in the conduct and monitoring of clinical trials. The DSMB members have been selected by the sponsor, after consultation with the clinical trial investigators. The DSMB is responsible for oversight of study safety considerations and for safeguarding the safety of clinical trial population through assessment of the trial safety data. On the basis of their review, the DSMB can conclude one of the following actions: continue, modify or discontinue the study.

A validated electronic data collection system (eCRF) will collect the clinical data (Castor EDC, XX). A GDPR-validated transmission system will be implemented to transfer the data to the core laboratory and DSMB for further assessment. An independent radiologist will function as imaging corelab. Patient imaging will be assessed on AAA diameter (multi-planar reconstruction, double oblique measured) and presence of possible subsequent endoleak (all types).

A clinical monitoring plan, based on risk assessments and focussed on the primary and on the secondary objectives, is in place at the sponsor’s site. This will be executed during the conduct of the study. Clinical data should be retained at the site for 15 years and at the sponsor’s premises for a minimum of 2 years after completion of the clinical study. Trial data can be accessed upon request.

Dissemination of the feasibility trial results will be achieved through submission to international peer-reviewed journals and presentations at international conferences.

## supplementary material

10.1136/bmjopen-2023-082380 online supplemental file 1

## References

[1] Dijkstra ML, Zeebregts CJ, Verhagen HJM (2020). Incidence, natural course, and outcome of type II endoleaks in infrarenal endovascular aneurysm repair based on the ENGAGE registry data. J Vasc Surg.

[2] Guo Q, Du X, Zhao J (2017). Prevalence and risk factors of type II endoleaks after endovascular aneurysm repair: a meta-analysis. PLoS ONE.

[3] Lo RC, Buck DB, Herrmann J (2016). Risk factors and consequences of persistent type II endoleaks. J Vasc Surg.

[4] Seike Y, Matsuda H, Shimizu H (2022). Nationwide analysis of persistent type II endoleak and late outcomes of endovascular abdominal aortic aneurysm repair in Japan: a propensity-matched analysis. Circulation.

[5] Wang Y, Yuan F, Bai Y (2022). Natural history and influence on long-term outcomes of isolated type II endoleak after endovascular aneurysm repair: a 10-year experience at a single center. Rev Cardiovasc Med.

[6] Dosluoglu HH, Rivero M, Khan SZ (2019). Pre-emptive nonselective perigraft aortic SAC embolization with coils to prevent type II endoleak after endovascular aneurysm repair. J Vasc Surg.

[7] Harry Yu HY, Lindström D, Wanhainen A (2019). Pre-emptive embolization of aortic side branches prior to endovascular aortic repair to prevent type II endoleaks: a systematic review and meta-analysis. Eur J Vasc Endovasc Surg.

[8] Wu Y, Yin J, Hongpeng Z (2022). Systematic review and network meta-analysis of pre-emptive embolization of the aneurysm sac side branches and aneurysm sac coil embolization to improve the outcomes of endovascular aneurysm repair. Front Cardiovasc Med.

[9] Branzan D, Geisler A, Steiner S (2021). Type II endoleak and aortic aneurysm sac shrinkage after preemptive embolization of aneurysm sac side branches. J Vasc Surg.

[10] Nakayama H, Toma M, Kobayashi T (2023). Abdominal aortic aneurysm shrinkage up to 2 years following endovascular repair with pembolization for preventing type II endoleak: a retrospective single center study. Ann Vasc Surg.

[11] Mathlouthi A, Guajardo I, Al-Nouri O (2021). Prophylactic aneurysm embolization during EVAR is safe, improves sac regression and decreases the incidence of type II Endoleak. Ann Vasc Surg.

[12] Rokosh RS, Chang H, Butler JR (2022). Prophylactic sac outflow vessel embolization is associated with improved sac regression in patients undergoing endovascular aortic aneurysm repair. J Vasc Surg.

[13] Piazza M, Squizzato F, Zavatta M (2016). Outcomes of endovascular aneurysm repair with contemporary volume-dependent sac embolization in patients at risk for type II endoleak. J Vasc Surg.

[14] Mascoli C, Faggioli G, Fenelli C (2019). Coil embolization of abdominal aortic aneurysm with a volume-tailored concentration in the prevention of persistent type II endoleak. Eur J Vasc Endovasc Surg.

[15] Mascoli C, Freyrie A, Gargiulo M (2016). Selective intra-procedural AAA sac embolization during EVAR reduces the rate of type II endoleak. Eur J Vasc Endovasc Surg.

[16] Fabre D, Fadel E, Brenot P (2015). Type II endoleak prevention with coil embolization during endovascular aneurysm repair in high-risk patients. J Vasc Surg.

[17] Landsman TL, Bush RL, Glowczwski A (2016). Design and verification of a shape memory polymer peripheral occlusion device. J Mech Behav Biomed Mater.

[18] Pleban E, Michalak J, Iwanowski J (2021). The dilemma after sealing an endovascular aortic aneurysm - three ways out. Zentbl Chir.

[19] Miranda I, Souza A, Sousa P (2021). Properties and applications of PDMS for biomedical engineering: a review. J Funct Biomater.

[20] van der Steenhoven TJ, Bosman WMPF, Tersteeg C (2012). Thrombogenicity of a new injectable biocompatible elastomer for aneurysm exclusion, compared to expanded polytetrafluoroethylene in a human ex vivo model. Eur J Vasc Endovasc Surg.

[21] Bosman W-MPF, van der Steenhoven TJ, Suárez DR (2010). The effect of injectable biocompatible elastomer (PDMS) on the strength of the proximal fixation of endovascular aneurysm repair grafts: an in vitro study. J Vasc Surg.

[22] Bosman W-MPF, Hinnen J-W, van der Steenhoven TJ (2011). Treatment of types II-IV endoleaks by injecting biocompatible elastomer (PDMS) in the aneurysm SAC: an in vitro study. J Endovasc Ther.

[23] Bosman W-MPF, van der Steenhoven TJ, Hinnen J-W (2010). Aortic customize: a new alternative endovascular approach to aortic aneurysm repair using injectable biocompatible elastomer. J Vasc Surg.

[24] Bosman WMPF, Vlot J, van der Steenhoven TJ (2010). Aortic customize: an in vivo feasibility study of a percutaneous technique for the repair of aortic aneurysms using injectable elastomer. Eur J Vasc Endovasc Surg.

[25] Doorschodt BM, Brom HL, de Vries AC (2016). In vivo evaluation of customized aortic repair using a novel survival model. Eur J Vasc Endovasc Surg.

[26] Smorenburg SPM, Lely RJ, Kelckhoven B-J van (2023). Initial clinical experience with aneufix injectable biocompatible elastomer for translumbar embolization of type II endoleaks. J Endovasc Ther.

[27] Güntner O, Zeman F, Wohlgemuth WA (2014). Inferior mesenteric arterial type II endoleaks after endovascular repair of abdominal aortic aneurysm: are they predictable. Radiology.

[28] Chan A-W, Tetzlaff JM, Gøtzsche PC (2013). SPIRIT 2013 explanation and elaboration: guidance for protocols of clinical trials. BMJ.

