# Supplementary material for: Aortic aneurysm sac filling with AneuFix injectable polymer during endovascular aneurysm repair: feasibility and safety trial study protocol
Source: BMJ Open. 2024 Jul 15;14(7):e082380. doi: 10.1136/bmjopen-2023-082380 (PMC11253768; doi:10.1136/bmjopen-2023-082380)
Supplement: online supplemental file 1 [file bmjopen-14-7-s001.pdf]

1 Supplementary Material: Safety reporting

1.1 Temporary halt for reasons of subject safety

In accordance with section 10, subsection 4, of the WMO, the sponsor will suspend the study if there is sufficient ground that continuation of the study will jeopardise subject health or safety. The sponsor will notify the accredited METC without undue delay of a temporary halt including the reason for such an action. The study will be suspended pending a further positive decision by the accredited METC. The investigator will take care that all subjects are kept informed.

1.2 AEs, SAEs and Device Related Effects

1.2.1 Adverse events (AEs)

Adverse events are defined as untoward medical occurrence, unintended disease or injury, or untoward clinical signs (including abnormal laboratory findings) in subjects, users or other persons, whether or not related to the investigational medical device and whether anticipated or unanticipated.

Note1: This definition includes events related to the investigational device or the comparator.
Note2: This definition includes events related to the procedures involved.
Note3: For users or other persons this definition is restricted to events related to the investigational medical devices or comparators

Vascular related adverse events: An AE that relates qua origin or cause to the human vasculature or the blood. More specifically, these AEs are typically related to the AAA, the vascular wall, the EVAR procedure and post-EVAR complications (e.g. endoleak).

All vascular related adverse events reported spontaneously by the subject or observed by the investigator or his staff will be recorded.

1.2.2 Serious adverse events (SAEs)

A serious adverse event is an AE that led to any of the following:
(a) death;
(b) a serious deterioration in the health of the subject, users or other persons as defined by one or more of the following:

- a life-threatening illness or injury, or
- permanent impairment of a body structure or a body function including chronic diseases, or
- hospitalisation or prolongation of patient hospitalisation, or
- medical or surgical intervention to prevent life-threatening illness, or injury or permanent impairment to a body structure or a body function

(c) foetal distress, foetal death, a congenital abnormality, or birth defect including physical or mental impairment.
Note: Planned hospitalization for a pre-existing condition, or a procedure required by the CIP, without serious deterioration in health, is not considered a serious adverse event.

1.2.3 Device Related Effects

ADE: Adverse Device Effect: AE related to the use of an investigational medical device.

SADE: Serious Adverse Device Effect: ADE that has resulted in any of the consequences characteristic of a SAE.

USADE: Unanticipated Serious Adverse Device effect: SADE which by its nature, incidence, severity or outcome has not been identified in the current version of the risk analysis report.

Anticipated SADE: A SADE that is listed as a potential issue in the Risk Analysis of the company (see IMDD).

Device Deficiency: Any inadequacy in the identity, quality, durability, reliability, safety or performance of the investigational device. This includes malfunctions, use errors, and inadequate labelling. Device deficiencies that did not lead to an adverse event but could have led to a medical occurrence:

a) if either suitable action had not been taken,
b) if intervention had not been made, or
c) if circumstances had been less fortunate

This clinical investigation is executed in accordance with ISO 14155 (2020) and MDR (EU) 2017/745.

All vascular related adverse events; ADEs, SAEs, SADEs and USADEs shall be documented in a timely manner throughout the clinical investigation.

From the regulatory reportable AEs, sufficient information will be obtained so as to permit 1) an adequate determination of the outcome of the event (i.e. whether the effect should be classified as an SAE) and; 2) an assessment of the causal relationship between the AE and the investigational devices. The following information will be collected for those AEs that require regulatory reporting:

- Title of Event
- Start date of event
- Intensity of event
- Frequency
- Outcome
- Relationship to Device/Procedure
- Seriousness Criteria
- Action Taken

Instructions will be given to the local study team with respect to the contact person at the Sponsor in case of observation of SAEs, SADEs and USADEs.

Vascular related AEs occurring in any patient, who signed the informed consent form, will need to be documented between the time the subject signed the informed consent and the time the subject departs the study after the month 24 follow-up phone call.

If a patient withdraws from the study prior to AneuFix treatment or is considered a screen failure, AEs until that study point need to be reported but there are no further follow-up requirements. If the patient was enrolled in the study but withdrew consent after AneuFix treatment, reportable AE's will be documented minimally during a one-month (4 weeks) follow-up period.

Safety data will be reviewed by the Data Safety Monitoring Board, the Coordinating Investigator and a representative of the sponsor to decide on complete documentation, device relatedness and impact on risk assessment. Recommendations to reduce the risk will be made and documented. Appropriate measures will be implemented by the sponsor as based on the recommendations received.

During any time of the process, attention should be paid to signals that can indicate a serious health threat. Signals from adverse events or device deficiencies that might indicate a serious health threat can be detected by either the sponsor or principal investigator but are evaluated by the sponsor.

For the SAE regulatory reporting requirements are summarized in the below table.

Member State	Ethics Committee Requirements	Competent Authority Requirements	Submission format
	Reporting SAEs into: www.toetsingonline.nl<br><br>Annual report to be submitted to EC	**<u>CCMO</u>**<br><br>SAEs with risk of death, serious injury or illness and require immediate action to treat the patient, including SAEs that lead to (temporary) discontinuation of the enrolment of patients, discontinuation of the study or adaptation in medical device.:<br><br>**Immediately (within 2 business days, no later than 4 calendar days)**	**MEDDEV Summary Tabulation**

		Calculated risks are excluded from these timelines as long as (temporary) discontinuation of enrolment or study or adaptation in device is not applicable	
		All other reportable events defined as:<br><br>1) any SAE not meeting above-mentioned criteria<br>2) any Device Deficiency that might have led to a SAE if:<br>- suitable action had not been taken or<br>- intervention had not been made or<br>- if circumstances had been less fortunate<br>3) new findings/updates in relation to already reported events.<br><br>**Quarterly**	**MEDDEV Summary Tabulation** (included as Appendix C)
		As per 26-May-2021<br><br>All reportable events which indicate an imminent risk of death, serious injury, or serious illness and that requires prompt remedial action for other patients/subjects, users or other persons or a new finding to it<br><br>**Immediately, but not later than 2 calendar days after awareness by sponsor of a new reportable event or of new information in relation with an already reported event.**	**Clinical Investigation Summary Safety Report Form** (included as Appendix D)<br><br>Submit in ToetsingOnline for dossier# NL73223.029.20
		As per 26-May-2021<br><br>Any other reportable events or a new finding/update to it:<br><br>**Immediately, but not later than 7 calendar days following the date of awareness by the sponsor of the new reportable event or of new information in relation with an already reported event.**	**Clinical Investigation Summary Safety Report Form** (included as Appendix D)<br><br>Submit in ToetsingOnline for dossier# NL73223.029.20

1.3 Follow-up of adverse events

During the period of the investigation the handling of adverse events will occur per the guidelines given by the European Commission on clinical investigations[1]. As such new findings/updates in relation to already reported events are considered reportable events.
At all visits vascular related AE's, ADEs, SAEs, (U)SADEs, and new findings/updates in relation to already reported events must be reported to the sponsor. In case of death or SAE's requiring urgent medical interventions: by sending the completed SAE report form to the safety team or directly into the electronic database within 24 hours of the investigator becoming aware of the SAE.
The investigator should institute appropriate therapeutic and follow-up measures in accordance with good medical practice but should notify the medical monitor of such actions and record them in the patient's case report form.

[1] http://ec.europa.eu/health/medical-devices/files/meddev/2_7_3_en.pdf

All vascular related adverse events, ADEs, SAEs, SADEs and USADEs will be followed-up until they are resolved or for one month (4 weeks) after the patient's participation in the clinical investigation ends.

1.4 Data Safety Monitoring Board (DSMB)

The DSMB is an independent group consisting of expert clinicians, who collectively have experience in the management of patients with abdominal aneurysm treatment and in the conduct and monitoring of clinical trials. The DSMB members have been selected by the sponsor, after consultation with the clinical trial principal investigators.
The DSMB is responsible for oversight of study safety considerations and for safeguarding the safety of clinical trial population through assessment of the trial safety data.
The DSMB will receive and review the progress of this trial, will assess the safety aspects based on the review of reported adverse events, and provide recommendations to the sponsor to (dis)continue with the study. A scheduled review of peri-procedural safety data will be conducted after the first 2 and 4 patients are treated with Aneufix.

On the basis of their review, the DSMB can conclude one of the following actions:

- Continue the study according to the protocol and any related amendments.
- Modify the study protocol. Modifications may include, but are not limited to, changes in inclusion/exclusion criteria, frequency of visits of safety monitoring, alterations in study procedures, changes in duration of observation, and follow up.
- Discontinue the study (with provisions for orderly discontinuation in accord with good medical practice).

The conclusion of the review will be formulated as a recommendation to the Sponsor.

In order to allow the DSMB make appropriate reviews and decisions on the safety, access to the coded CT-scans (procedure and possible FU sessions) has to be granted and assured. A GDPR-compliant transmission system is used for this access.

The advice(s) of the DSMB will only be sent to the sponsor of the study. Should the sponsor decide not to fully implement the advice of the DSMB, the sponsor will send the advice to the reviewing METC, including a note to substantiate why (part of) the advice of the DSMB will not be followed.

The activities of the DSMB are detailed in a separate Charter 'Data Safety Monitoring Board Charter'.
