# Supplementary material for: Aortic aneurysm sac filling with AneuFix injectable polymer during endovascular aneurysm repair: feasibility and safety trial study protocol
Source: BMJ Open. 2024 Jul 15;14(7):e082380. doi: 10.1136/bmjopen-2023-082380 (PMC11253768; doi:10.1136/bmjopen-2023-082380)
Supplement: Supplementary file 2 [file bmjopen-2023-082380.reviewer_comments.pdf]

PEER REVIEW HISTORY

BMJ Open publishes all reviews undertaken for accepted manuscripts. Reviewers are asked to complete a checklist review form (**http://bmjopen.bmj.com/site/about/resources/checklist.pdf**) and are provided with free text boxes to elaborate on their assessment. These free text comments are reproduced below.

ARTICLE DETAILS

**TITLE (PROVISIONAL)**	Aortic Aneurysm Sac Filling with AneuFix Injectable Polymer during Endovascular Aneurysm Repair: Feasibility and Safety Trial Study Protocol
**AUTHORS**	Smorenburg, Stefan; Lely, Rutger; Jacobs, Michael; Hoksbergen, Arjan

VERSION 1 – REVIEW

**REVIEWER**	Lo, Zhiwen<br>Tan Tock Seng Hospital, Vascular Surgery Service, Department of General Surgery
**REVIEW RETURNED**	10-Dec-2023

**GENERAL COMMENTS**	Thank you for your submission and kudos for an excellent study protocol. Looking forward to the results of your pivotal study. Your study brings to mind previous EVAS study, which you have briefly mentioned (Pg 5 lines 35). As we know, the adverse events for EVAS occured after 24 months. Hence would the authors consider the following 2 points:<br>- described further the differences between EVAS and AneuFix, so that lessons learnt from EVAS' failure will not be repeated<br>- consider extension of follow-up from the current 24 months to 36 months.<br>Thank you.

**REVIEWER**	Ni, Leng<br>Peking Union Medical College Hospital, Department of Vascular Surgery
**REVIEW RETURNED**	29-Apr-2024

**GENERAL COMMENTS**	This study aims to investigate the safety and feasibility of prophylactic aortic aneurysm sac filling with AneuFix biocompatible injectable polymer during EVAR in patients at high risk for type II endoleak. Regarding to the follow-up method, you plan to use CTA to evaluate whether the tyep II endoleak exist or not. However, in most situation, CTA is not an accurate method to evaluate type II endoleak, especially for some obscure endoleak, DSA is required to varify the endoleak.

VERSION 1 – AUTHOR RESPONSE

Reviewer: 1
Dr. Zhiwen Lo, Tan Tock Seng Hospital
Comments to the Author:
Thank you for your submission and kudos for an excellent study protocol. Looking forward to the

results of your study.
Dear Dr. Zhiwen Lo,
Thank you very much for your time and effort for reviewing our study protocol. We have answered your comments below.
Your study brings to mind previous EVAS study, which you have briefly mentioned (Pg 5 lines 35). As we know, the adverse events for EVAS occurred after 24 months. Hence would the authors consider the following 2 points:
1. Describe further the differences between EVAS and AneuFix, so that lessons learnt from EVAS' failure will not be repeated
We fully agree and have added the following to the manuscript:
“ The crucial difference between EVAR with active sac filling and EVAS is the approach to sealing the aneurysm and stent-graft fixation. EVAS does not involve fixation above or below the aneurysm. Instead, it focuses on sealing off the aneurysm sac itself using polymer-filled endobags that conform to the sac shape. In contrast, EVAR with active sac filling utilises a standard EVAR stent graft, which achieves proximal and distal seal above and below the aneurysm in relatively healthy aortoiliac tissue, effectively isolating the aneurysm from blood flow. Adjacent to this, the AAA sac is filled around the standard EVAR stent graft.”
(Introduction, page 4).
2. Consider extension of follow-up from the current 24 months to 36 months. Thank you.
Indeed, it is important to keep track of the patient after 24 months. We have added the following to the manuscript:
“After the 24 months follow-up, patients have reached study completion however, patients will remain in follow-up per local hospital protocol typically involving yearly duplex or CT assessment.” (Methods, p:11).
Reviewer: 2
Dr. Leng Ni, Peking Union Medical College Hospital
Comments to the Author:
This study aims to investigate the safety and feasibility of prophylactic aortic aneurysm sac filling with AneuFix biocompatible injectable polymer during EVAR in patients at high risk for type II endoleak.
Dear Dr. Leng Ni,
Thank you very much for your time and effort for reviewing our study protocol. We have answered your comments below.
Regarding to the follow-up method, you plan to use CTA to evaluate whether the type II endoleak exist or not. However, in most situation, CTA is not an accurate method to evaluate type II endoleak, especially for some obscure endoleak, DSA is required to verify the endoleak.
In the case of a growing aneurysm during follow-up and a non-conclusive CTA we will use other imaging modalities such as MRA to detect and classify the endoleak during follow-up. If there still remains doubt concerning the endoleak type, we perform DSA procedure.

VERSION 2 – REVIEW

**REVIEWER**	Lo, Zhiwen<br>Tan Tock Seng Hospital, Vascular Surgery Service, Department of General Surgery
**REVIEW RETURNED**	20-Jun-2024

**GENERAL COMMENTS**	Thank you for your revision

**REVIEWER**	Ni, Leng<br>Peking Union Medical College Hospital, Department of Vascular Surgery
**REVIEW RETURNED**	23-Jun-2024

**GENERAL COMMENTS**	No special comments.
